# Myocardial Function during Ventilation with Lower versus Higher Positive End-Expiratory Pressure in Patients without ARDS

**DOI:** 10.3390/jcm11092309

**Published:** 2022-04-21

**Authors:** Anna Geke Algera, Charalampos Pierrakos, Michela Botta, Claudio Zimatore, Luigi Pisani, Pieter-Roel Tuinman, Lieuwe D. J. Bos, Wim K. Lagrand, Marcello Gama de Abreu, Paolo Pelosi, Ary Serpa Neto, Marcus J. Schultz, Thomas G. V. Cherpanath, Frederique Paulus

**Affiliations:** 1 Department of Intensive Care, Amsterdam University Medical Centers Location University of Amsterdam, 1105 AZ Amsterdam, The Netherlands; charalampos_p@hotmail.com (C.P.); m.botta@amsterdamumc.nl (M.B.); claudiozimatore@gmail.com (C.Z.); luigipisani@gmail.com (L.P.); l.d.bos@amsterdamumc.nl (L.D.J.B.); w.k.lagrand@amsterdamumc.nl (W.K.L.); marcus.j.schultz@gmail.com (M.J.S.); t.g.cherpanath@amsterdamumc.nl (T.G.V.C.); f.paulus@amsterdamumc.nl (F.P.); 2 Laboratory of Experimental Intensive Care and Anesthesiology (LEICA), Amsterdam University Medical Centers Location University of Amsterdam, 1105 AZ Amsterdam, The Netherlands; 3 Department of Intensive Care, Brugmann University Hospital, Université Libre de Bruxelles, 1020 Brussel, Belgium; 4 Section of Anesthesia and Intensive Care, Department of Emergency and Organ Transplantation, University of Bari Aldo Moro, 70124 Bari, Italy; 5 Mahidol-Oxford Tropical Medicine Research Unit (MORU), Mahidol University, Bangkok 10400, Thailand; 6 Department of Intensive Care & Research VUmc Intensive Care (REVIVE), Amsterdam University Medical Centers Location Vrije Universiteit Amsterdam, 1081 HV Amsterdam, The Netherlands; p.tuinman@amsterdamumc.nl; 7 Department of Anesthesiology and Intensive Care, University Hospital Carl Gustav Carus, 01307 Dresden, Germany; mgabreu@uniklinikum-dresden.de; 8 Department of Surgical Sciences and Integrated Diagnostics, IRCCS San Martino Policlinico Hospital, University of Genoa, 16132 Genoa, Italy; ppelosi@hotmail.com; 9 Australian and New Zealand Intensive Care Research Centre (ANZIC-RC), School of Public Health and Preventive Medicine, Monash University, Melbourne, VIC 3004, Australia; ary.serpaneto@monash.edu; 10 Department of Critical Care Medicine, Melbourne Medical School, Austin Hospital, University of Melbourne, Heidelberg, VIC 3084, Australia; 11 Data Analytics Research and Evaluation (DARE) Centre, Austin Hospital, Heidelberg, VIC 3084, Australia; 12 Department of Intensive Care Medicine, Hospital Israelita Albert Einstein, Sao Paulo 05652-900, Brazil; 13 Nuffield Department of Medicine, Oxford University, Oxford OX3 7BN, UK; 14 Center of Expertise Urban Vitality, Faculty of Health, Amsterdam University of Applied Sciences, 1095 DZ Amsterdam, The Netherlands

**Keywords:** ARDS, ICU, MPI, myocardial function, PEEP, mechanical ventilation

## Abstract

The aim of this study was to investigate whether lower PEEP (positive end-expiratory pressure) had beneficial effects on myocardial function among intensive care unit (ICU) patients without acute respiratory distress syndrome (ARDS) compared to higher PEEP. In this pre-planned substudy of a randomized controlled trial (RELAx), comparing lower to higher PEEP, 44 patients underwent transthoracic echocardiography. The exclusion criteria were known poor left ventricular function and severe shock requiring high dosages of norepinephrine. To create contrast, we also excluded patients who received PEEP between 2 cmH2O and 7 cmH2O in the two randomization arms of the study. The primary outcome was the right ventricular myocardial performance index (MPI), a measure of systolic and diastolic function. The secondary outcomes included systolic and diastolic function parameters. A total of 20 patients were ventilated with lower PEEP (mean ± SD, 0 ± 1 cmH2O), and 24 patients, with higher PEEP (8 ± 1 cmH2O) (mean difference, −8 cmH2O; 95% CI: −8.1 to −7.9 cmH2O; *p* = 0.01). The tidal volume size was low in both groups (median (IQR), 7.2 (6.3 to 8.1) versus 7.0 (5.3 to 9.1) ml/kg PBW; *p* = 0.97). The median right ventricular MPI was 0.32 (IQR, 0.26 to 0.39) in the lower-PEEP group versus 0.38 (0.32 to 0.41) in the higher-PEEP group; the median difference was –0.03; 95% CI: −0.11 to 0.03; *p* = 0.33. The other systolic and diastolic parameters were similar. In patients without ARDS ventilated with a low tidal volume, a lower PEEP had no beneficial effects on the right ventricular MPI.

## 1. Introduction

Mechanical ventilation, the most frequently applied strategy in the intensive care unit (ICU), is a potentially harmful intervention [1]. Protective ventilation, a strategy aiming at reducing the intensity of mechanical stimulation on lung tissue, is often used to mitigate the detrimental effects of mechanical ventilation [2]. While the protective role of a lower tidal volume (VT) is well defined, uncertainty remains regarding the protective effects of the positive end-expiratory pressure (PEEP), particularly in patients without acute respiratory distress syndrome (ARDS) [3]. Furthermore, over the last few years, there has been a noticeable increase in the use of higher PEEP in ICU patients without ARDS [4]. Therefore, the REstricted versus Liberal positive end-expiratory pressure in patients without ARDS (RELAx) trial investigated the impact of using lower PEEP (the lowest possible PEEP level between 0 and 5 cmH2O) compared with using higher PEEP (8 cmH2O) in patients without ARDS [5].

PEEP is well-known to cause significant hemodynamic changes that could lead to a decreased cardiac index [6]. A higher PEEP may increase intrathoracic pressure, leading to an increase in the right ventricular afterload, decreased venous return and decreased left and right ventricular contractility [7]. Clinical studies that evaluated the effects of PEEP on right heart preload [8,9] and on right ventricle contractility and afterload [10,11,12] showed a heterogeneous response depending on global heart function and the levels of PEEP applied. Importantly, these studies investigated the effects of PEEP levels well above 10 cmH2O, a level that is usually not applied in patients without ARDS. Experimental studies in different animal models without ARDS showed ventilation with higher PEEP to have a negative effect on cardiac output compared to ventilation with lower PEEP [13,14,15]. However, these investigations were heterogeneous in their design and outcomes [15].

It remains uncertain if ventilation with a PEEP of 8 cmH2O could negatively impact cardiac function in ICU patients without ARDS. Therefore, this study was conducted to compare lower versus higher PEEP on right and left ventricular function in patients without ARDS, assessed by echocardiography between 24 and 48 h after the start of invasive ventilation. We hypothesized that lower PEEP has beneficial effects on right ventricular systolic and diastolic function compared to higher PEEP with the use of low tidal volumes.

## 2. Materials and Methods

### 2.1. Study Design and Setting

The RELAx trial (clinicaltrials.gov accessed on 20 January 2022, trial number NCT03167580) was a national, multicenter, randomized clinical trial in invasively ventilated ICU patients without ARDS [5]. Patients were randomized to a ventilation strategy with lower PEEP, in which the PEEP was titrated from 5 cmH2O to the lowest level at which oxygenation remained satisfactory, versus a ventilation strategy with higher PEEP, in which the PEEP was set at 8 cmH2O. In the recently published RELAx trial, a lower-PEEP strategy was noninferior to a higher-PEEP strategy with regard to the number of ventilator-free days at day 28; these findings supported the use of lower PEEP in patients without ARDS.

We performed a single-center transthoracic echocardiography substudy of RELAx patients enrolled at the Amsterdam University Medical Center, location AMC, who were mechanically ventilated for 24 to 48 h according to the study protocol. Within this timeframe of 24 to 48 h of mechanical ventilation according to the RELAx protocol, we assessed and compared changes in cardiac function as measured by transthoracic echocardiography in response to the compared ventilation strategies. This timeframe was chosen to obtain informed consent of the legal representative, it enabled a relatively stable setting in which the echocardiogram could be performed and allowed sufficient time for the required PEEP titration as targeted in the protocol. The institutional review board of the Amsterdam University Medical Center approved this substudy (2017_074#B2018435, 18 July 2018, Amsterdam, the Netherlands), and deferred informed consent was obtained from a legal representative for this substudy, as part of the parent study RELAx.

The parent study enrolled patients who were expected to be ventilated for more than 24 h; the exclusion criteria included the presence of ARDS, COPD GOLD class III or IV, restrictive pulmonary disease, ongoing cardiac ischemia and severe untreatable anemia. The exclusion criteria were known poor left ventricular function (an ejection fraction less than or equal to 30%), severe shock requiring norepinephrine greater than or equal to 0.5 µg/kg/min, and ventilation with PEEP greater than 2 cmH2O in the lower-PEEP group and less than 7 cmH2O in the higher-PEEP group.

Transthoracic echocardiography images were recorded using a commercially available ultrasound system with a 2–5 MHz sector probe (Vivid 9 Dimension; GEVindmed Ultrasound AS, Norway). The images were continuously and digitally stored according to the local standard protocol.

### 2.2. Study Protocol

The full details of the study methods, including the ventilation strategies, have been described previously [16]. Briefly, within 1 h of the initiation of ventilation in the ICU, the patients were randomized in a 1:1 ratio to a ventilation strategy using lower or higher PEEP. The local investigators randomized patients using a central, dedicated, password-protected, encrypted, web-based automated randomization system (SSL-encrypted website with the ALEA software (version 16), TenALEA Consortium, Amsterdam, the Netherlands). Randomization was conducted using random block sizes with a maximum of 8 patients. The attending nurses and physicians were not blinded to the intervention. The patients randomized to the lower-PEEP group started with 5 cmH2O, and every 15 min, the PEEP was down-titrated by 1 cmH2O to a minimum of 0 cmH2O. For the patients assigned to the higher-PEEP group, the PEEP was set to 8 cmH2O. If the SpO_2_ or PaO_2_ dropped below 92%, or below 60 mm Hg for more than 5 min, the FiO_2_ was increased to the maximal 0.6 before the PEEP was increased in steps of 1 cmH2O up to 5 cmH2O (lower-PEEP group) or up to more than 8 cmH2O (higher-PEEP group).

Before the transthoracic echocardiography, the hemodynamic and respiratory data of the patients were recorded. All the ventilator settings and drug doses remained unaltered during the approximately 30 min required for transthoracic echocardiography. If arterial blood gas data were collected within 4 h of the transthoracic echocardiography, these data were obtained from the electronic patient data system. Skin electrodes were attached to generate a continuous cardiac rhythm on the echocardiogram with a minimum recording of three cardiac cycles in the case of a sinus rhythm, or five cardiac cycles in the case of atrial fibrillation according to guidelines [17].

The right ventricular myocardial performance index was the primary endpoint of the study. The myocardial performance index was calculated from tissue Doppler imaging by adding the isovolumetric contraction time to the isovolumetric relaxation time and then dividing the sum by the ejection time. The secondary endpoints included the left ventricular myocardial performance index, and various systolic and diastolic echocardiographic parameters (an overview of all the other obtained variables are provided in Supplement S1).

The images were obtained by examiners trained in echocardiographic procedures in critically ill patients (C.Z., M.B., L.P., A.A. and C.P.). Thereafter, the images were analyzed offline using automated function imaging software (EchoPAC; GE Vingmed, Norway) by a blinded investigator.

### 2.3. Statistical Analysis

Based on the results of a recent study in a comparable patient cohort [18], we expected that 18 patients per PEEP group would be sufficient to achieve a power of 80% with a two-sided significance level of 0.05 to detect a 0.12 difference in the myocardial performance index of the right heart. The sample size was increased by 20% to correct for dropouts (i.e., if the myocardial performance index could not be determined from transthoracic echocardiography due to insufficient windows), meaning that a total of 44 patients were required.

Continuous variables were compared between the PEEP groups using the independent-samples *t*-test in the case of a normal distribution; otherwise, the Mann-Whitney U test was used. Categorical variables were compared between the PEEP groups using the chi-square test. Categorical data are reported as numbers with percentages in parentheses. Continuous data are reported as means with their standard deviations (SDs) in the case of a normal distribution; otherwise, medians with their interquartile ranges (IQRs) are provided. Comparisons are shown with the mean difference and the 95% confidence interval (CI) from the independent-samples *t*-test in normally distributed cases; otherwise, the Hodges-Lehmann estimate of the median difference and 95% CI were used.

All the analyses were performed in R through the RStudio interface (available online: http://www.r-project.org, R version 3.3.1, accessed on 2 February 2022). A two-sided *p* value under 0.05 was considered statistically significant.

## 3. Results

**Figure 1:**
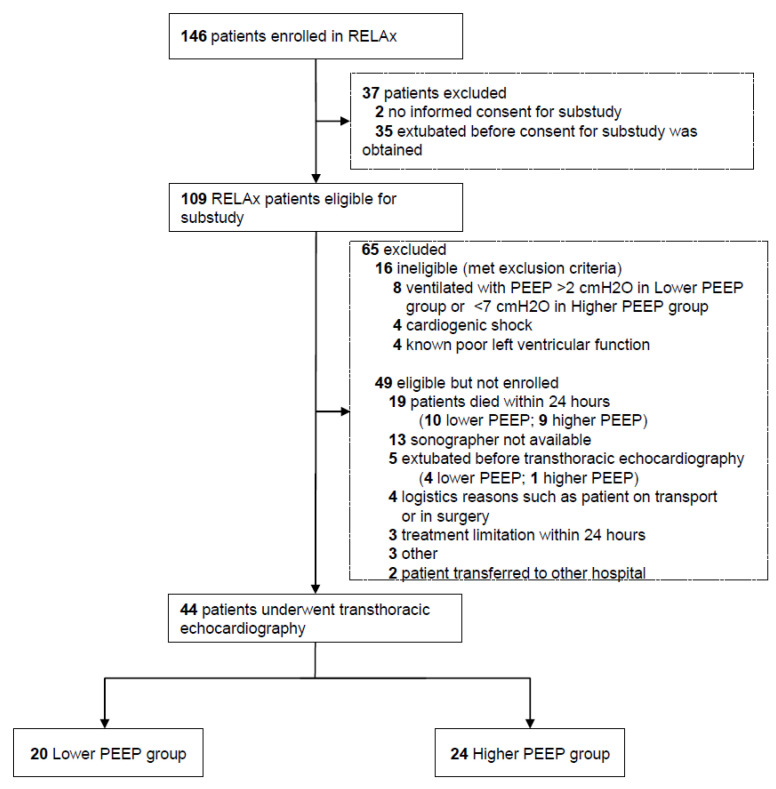
Flow of patients. Abbreviations: PEEP, positive end-expiratory pressure.

From July 2018 through December 2019, 146 patients were enrolled in the RELAx trial in our center. After the exclusion of patients not eligible for this substudy, 109 patients remained suitable for participation. Of these, 65 patients were excluded (16 met the exclusion criteria, and 49 were eligible but not enrolled), leaving 44 patients who underwent a transthoracic echocardiography examination. Data for the 44 patients, 20 patients allocated to lower PEEP and 24 patients allocated to higher PEEP, were analyzed (Figure 1).

### 3.1. Baseline Characteristics

The baseline characteristics are presented in Table 1. Of the enrolled patients, 75% were admitted to the ICU for a medical reason. The most frequent reason for invasive ventilation was respiratory failure (29.5%). The majority of the patients were ventilated by pressure support ventilation upon transthoracic echocardiography examination. All but five patients were in sinus rhythm, with three patients in atrial fibrillation in the lower-PEEP group and two patients in atrial fibrillation in the higher-PEEP group. The incidence and rates of infusion of vasopressors were comparable between the two groups. Only one patient received the infusion of an inotrope, at a rather low dose. More than one-third of the patients received sedation. The fluid balance on the day of transthoracic echocardiography examination was higher in the higher-PEEP group.

### 3.2. Respiratory and Hemodynamic Parameters

The respiratory and hemodynamic parameters are presented in Table 2. Patients were invasively ventilated according to the study protocol for a median of 36 (IQR: 27 to 46) hours at the moment of transthoracic echocardiography examination. The patients in the lower-PEEP group received a mean PEEP of 0 ± 1 cmH2O, and the patients in the higher-PEEP group received a mean PEEP of 8 ± 1 cmH2O (mean difference, –8 cmH2O; 95% CI, –8 to –8 cmH2O; *p* < 0.01). Accordingly, the maximum airway pressure was lower in the patients ventilated with lower PEEP compared with those ventilated with higher PEEP (12 ± 4 versus 20 ± 4 cmH2O; mean difference, –8 cmH2O; 95% CI, –11 to –6 cmH2O; *p* < 0.01). The tidal volumes were similar for the two PEEP groups. The mean pH was higher in the lower-PEEP group; the mean hemoglobin was higher in the higher-PEEP group. The hemodynamic parameters did not differ between the two PEEP groups.

### 3.3. Echocardiographic Evaluation

Mitral or aortic valvopathy was present in none of the patients. There were no differences in the mean diameter of the vena cava inferior or in the distensibility index (1.6 ± 0.5 versus 1.9 ± 0.6 cm (*p* = 0.22), and 32 (17 to 160) % versus 23 (7 to 49) % (*p* = 0.21) in the lower- and higher-PEEP groups, respectively).

### 3.4. Right and Left Ventricular Systolic and Diastolic Function

The ventricular systolic and diastolic function parameters are presented in Table 3. The indicators of increased right ventricular pressure and volume overload were not different between the groups, with no differences in the right ventricular afterload as well.

The primary endpoint, the right ventricular myocardial performance index, could not be acquired in five patients, in four patients from the higher-PEEP group and in one patient from the lower-PEEP group. The left ventricular myocardial performance index was obtained in all 44 patients. The median right ventricular myocardial performance index was 0.32 (IQR, 0.26 to 0.39) in the lower-PEEP group versus 0.38 (0.32 to 0.41) in the higher-PEEP group; median difference, –0.03; 95% CI, –0.11 to 0.03; *p* = 0.33. The median left ventricular myocardial performance index was 0.41 (IQR, 0.37 to 0.49) in the patients ventilated with lower PEEP versus 0.45 (0.39 to 0.54) in the patients ventilated with higher PEEP; median difference, –0.02; 95% CI, –0.09 to 0.04; *p* = 0.35. No differences were found in any parameter for the systolic and diastolic function of the left or right ventricular between the PEEP groups (Table 3).

## 4. Discussion

This study shows that mechanical ventilation with lower PEEP in ICU patients without ARDS does not affect the right ventricular myocardial performance index compared to higher PEEP. The left ventricular myocardial performance index and other systolic and diastolic echocardiographic parameters were also not different between the two PEEP groups.

The detrimental effects of PEEP on the heart have been assessed extensively in patients with ARDS [9,19,20,21]. However, the effects of PEEP levels on cardiac function have not been assessed thoroughly in patients without ARDS, and this clinical study adds information on whether ventilation with a lower PEEP improves cardiac function. Increasing pressure at the end of expiration can affect heart function by changing the lung volume and intrathoracic pressure independently of ARDS presence [7]. Nevertheless, several pathophysiological factors can amplify detrimental PEEP effects on cardiac function in patients with ARDS such as decreased lung compliance, hypoxia and hypercapnia [22], which are not often present in patients without ARDS. In the current study, the patients in both PEEP groups had PaCO_2_ values within the normal range, no hypoxia was observed, and the respiratory system was within normal ranges. In addition, there was sufficient time for the correction of a possible preload decrease in the higher-PEEP group as illustrated by the higher fluid balance. In this study, we observed that decreasing PEEP in patients without ARDS has minor effects on the right heart afterload and right and left systolic and diastolic function.

The effects of PEEP on cardiac function in this study should be considered within the context of ventilation with a lower tidal volume. In a recent study, performed in a similar patient group, the right ventricular myocardial performance index was lower during ventilation with a lower tidal volume versus a higher tidal volume (0.41 vs. 0.64) [18]. In that study, the PEEP was 5 cmH2O in both study arms. The findings of the current study add to our understanding of the effects of mechanical ventilation on the right ventricle, by showing that a ventilation strategy that may increase lung strain (i.e., ventilation with a higher tidal volume and higher PEEP from the previous study) resulted in a higher right ventricular myocardial performance index compared to a ventilation strategy that causes less lung strain (i.e., ventilation with a lower tidal volume and lower PEEP from the current study) (myocardial performance index of 0.64 versus 0.32) [18]. This could mean that an increase in tidal volume may have a more pronounced detrimental effect on right ventricle functioning than an increase in PEEP, or that the detrimental effects of higher PEEP were nullified by the use of a lower tidal volume in the current study. This should be evaluated in future studies.

The myocardial performance index, obtained using tissue Doppler imaging, was chosen in this study to assess right heart function for several reasons. The MPI is a straightforward, reproducible indicator of both systolic and diastolic function and is relatively independent of preload and afterload, and therefore, possible differences in the loading conditions, such as the fluid balance, will have limited effects on this parameter [23].

Over the years, there has been a clear increase in the use of higher PEEP in patients with ARDS and in patients without ARDS [24], although the benefit of ventilation with higher PEEP for mortality was only demonstrated for patients with moderate and severe ARDS, while it resulted in a longer duration of ventilation in patients with mild ARDS according to a meta-analysis [25]. Previous studies investigating the effects of PEEP on cardiac function in ventilated patients with ARDS often used higher levels of PEEP > 10 cmH2O [8,18,19,20]; therefore, it remains uncertain if PEEP levels lower than the previously investigated levels could negatively affect cardiac function.

In this study, we investigated if decreasing the PEEP from 8 to 2 cmH2O in ventilated patients without ARDS had beneficial effects on cardiac function. The vena cava distensibility index in this study indicates that the included patients were adequately volume resuscitated. This is supported by the difference in fluid balance of 900 mL between the two groups at the moment of echocardiography. One could suggest that this may have mitigated the detrimental effects of ventilation with a higher level of PEEP on cardiac preload.

The strengths of this study are the fact that the effects of PEEP were evaluated in a heterogeneous population of ICU patients without ARDS, who were randomly divided into two PEEP groups. The protocol of this study was pre-published, and the transthoracic echocardiography data were analyzed by a blinded physician. The patients in both groups were well equilibrated, and the tidal volume was not different between the two PEEP groups. The examiners who performed the echocardiograms could not be blinded for group assignment, but the obtained images were further analyzed offline by a blinded investigator. This is the first clinical study to assess the effects of decreasing PEEP to the lowest possible level on cardiac function in invasively ventilated patients not having ARDS.

This study also has some limitations. First, we only used transthoracic echocardiography to evaluate cardiac function. Future studies should include additional parameters of cardiac function to strengthen the current findings. Second, we did not perform serial transthoracic echocardiographies. A comparison from before intubation or shortly after intubation would have been interesting. However, this was not possible due to the design of the study; the randomization for the parent study was conducted within one hour of the start of ventilation in the ICU, the PEEP titrations were started immediately after randomization, and we required informed consent from the legal representative for this study before an echocardiography could be performed. Certainly, right ventricular function in patients requiring prolonged ventilation can be affected by several other factors (e.g., sepsis and ARDS). On the other hand, 24 to 48 h of mechanical ventilation is sufficiently long for studying the effects of PEEP without the risk of such confounders. Third, we did not measure the pulmonary artery pressure and therefore could not determine the effects with lower or higher PEEP on the pulmonary artery pressure and, with that, the afterload of the right ventricle. Fourth, the right ventricular myocardial performance index is relatively pre-load-independent; however, we cannot exclude the possibility that some patients may have had atrial pressures of more than 15 mmHg, causing a pseudonormalization of this index [26]. Furthermore, the hemoglobin level was significantly lower in the lower-PEEP arm, which may have been caused by differences in fluid administration between the two groups. Fifth, the primary endpoint could not be obtained in 5 out of 44 patients due to insufficient windows, which could have influenced the results. Obtaining sufficient echocardiographic windows can be challenging in ventilated critically ill patients. For this reason, in the design of the study, we increased the sample size by 20% to account for this challenge. Sixth, to have a distinct separation between the two PEEP groups, we excluded the patients who were ventilated with a PEEP > 2 cmH2O in the lower-PEEP arm and with a PEEP < 7 cmH2O in the higher-PEEP arm in this study. This may have induced bias. In 24 patients, we could not perform transthoracic echocardiography because of early death or early weaning from the ventilator. This may also have induced bias. Seventh, although we did not find a significant difference in the myocardial performance index, the Hogdes-Lehmann estimator ranged from −0.11 to 0.03; therefore, we cannot exclude a possible minor effect of PEEP on the right ventricular MPI with certainty. Finally, the patients were enrolled in a single academic center, which could limit the generalizability of the findings.

## 5. Conclusions

In patients without ARDS ventilated with a low tidal volume, ventilation with lower PEEP had no beneficial effects on the right ventricle myocardial performance index when compared to ventilation with higher PEEP.

## Figures and Tables

**Table 1:** Baseline characteristics of the patients.

	Lower PEEP (*n* = 20)	Higher PEEP (*n* = 24)	*p* Value
Age, y	64 (56 to 72)	65 (59 to 70)	0.93
Female gender, No. (%)	10 (50)	8 (33)	0.34
Height, cm	172 ± 10	175 ± 7	0.25
Weight, cm	76 ± 16	83 ± 16	0.15
SOFA score ^a^	10.1 ± 2.9	9.6 ± 3.7	0.69
Reason for ICU admission, No. (%)			0.07
Elective surgery	4 (20)	0 (0)	
Urgent surgery	3 (15)	4 (16)	
Medical	13 (65)	20 (84)	
Reason for intubation, No. (%)			0.28
Respiratory failure	6 (30)	7 (29)	0.42
Pneumonia	2 (33)	3 (43)	
Cardiogenic pulmonary edema	2 (33)	1 (14)	
Sepsis	2 (33)	2 (29)	
COPD, gold I-II	0 (0)	1 (14)	
Cardiac arrest	1 (5)	5 (21)	
Planned postoperative ventilation	7 (35)	3 (12)	
Depressed level of consciousness	5 (25)	6 (25)	
Airway protection	1 (5)	3 (12)	
Ventilatory mode, No. (%)			0.33
Pressure-controlled	5 (25)	6 (25)	
Pressure support	15 (75)	15 (63)	
Adaptive support ventilation	0	3 (12)	
Sedation	5 (25)	13 (54)	0.09
Propofol	5 (25)	12 (50)	0.17
Midazolam	2 (10)	2 (8)	1.00
RASS ^b^	−3 (−3 to −1)	−4 (−5 to −3)	0.06
Vasopressors and inotropes			
Norepinephrine, No. (%)	5 (25)	7 (29)	0.98
Norepinephrine dose, μg kg^−1^ min^−1^	0.12 ± 0.11	0.13 ± 0.08	0.78
Milrinon, No. (%)	1 (5)	0 (0)	0.93
Milrinon dose, μg kg^−1^ min^−1^	0.17 (0.18 to 0.17)	0 (0)	1
Sinus rhythm, No. (%)	17 (85)	22 (85)	0.83
Fluid balance, mL	304 (−604 to 928)	1215 (−89 to 1944)	0.04

Data are provided as mean ± SD when normally distributed; otherwise, median (IQR) is used. Numbers are presented with (%). ^a^ SOFA score ranges from 0 to 24, with higher values indicating a more severe condition. ^b^ RASS scores range from −5 to +4. Abbreviations: SD, standard deviation; ICU, intensive care unit; IQR, interquartile range; PEEP, positive end-expiratory pressure; RASS, Richmond Agitation–Sedation Scale; SOFA, Sequential Organ Failure Assessment.

**Table 2:** Respiratory and hemodynamic parameters at transthoracic echocardiographic examination.

	Lower PEEP (*n* = 20)	Higher PEEP (*n* = 24)	Point Estimate of the Difference (95% CI)	* p * Value
Time, h ^a^	36 (27 to 46)	36 (27 to 46)	0 (−7 to 9)	0.76
Respiration				
PEEP, cmH2O	0 ± 1	8 ± 1	−8 (−8.1 to −7.9)	<0.01
Pmax, cmH2O	11.7 ± 4.1	20.1 ± 4.7	−8.4 (−11.1 to −5.6)	<0.01
FiO_2_, %	27 (24 to 35)	30 (24 to 34)	−3 (−4 to 4)	0.91
SpO_2_	97 (96 to 99)	98 (95 to 99)	−1 (−0.9 to 1.9)	0.45
V_T_/predicted body weight, mL/kg ^b^	7.22 (6.3 to 8.1)	7.02 (5.3 to 9.1)	0.20 (−1.4 to1.2)	0.97
RR, breaths/min =	19 ± 6	22 ± 6	−3 (−6 to 1)	0.12
Minute volume, L/min =	9. 5(8.1 to 10.8)	10.2 (8.7 to 13.1)	−0.5 (−3.5 to 0.3)	0.14
Laboratory				
pH	7.46 ± 0.05	7.42 ± 0.04	0.04 (0.01 to 0.07)	0.01
PaCO_2_, kPa	4.6 (4.1 to 5.2)	5.1 (4.7 to 5.5)	−0.5 (−0.9 to 0.1)	0.07
PaO_2_, kPa	10.8 (10.4 to 12.6)	11.0 (10.1 to 12.0)	-0.2 (−1.1 to 1.1)	0.82
Hemoglobin, mmol/L	6.3 ± 0.05	7.3 ± 0.04	−1 (−1.9 to −0.2)	0.01
Hemodynamics				
HR, mmHg	81 (67 to 100)	82 (66 to 105)	−1 (−18 to 10.9)	0.65
SBP, mmHg	138 ± 26	125 ± 29	13 (−4 to 29)	0.15
DBP, mmHg	63 ± 11	61 ± 12	2 (−4 to 9)	0.51
MAP, mmHg	88 ± 15	82 ± 15	6 (−2 to 15)	0.17

Data are provided as mean ± SD when normally distributed; otherwise, median (IQR) is used. Comparisons are shown with the point estimate of the mean or median difference, 95% CI and two-sided *p* value. ^a^ Time after randomization to the lower-PEEP or higher-PEEP strategy according to the RELAx trial. ^b^ Predicted body weight was calculated as 50 + 0.91 × (height [cm]–152.4) for men and 45.5 + 0.91 (height [cm]–152.4) for women. Abbreviations: DBP, diastolic blood pressure; FiO_2,_ fraction of inspired oxygen; Hb, hemoglobin; HR, heart rate; ICU, intensive care unit; IQR, interquartile range; MAP, mean arterial pressure; PaCO_2_, partial pressure of carbon dioxide; PaO_2_, partial pressure of arterial oxygen; PEEP, positive end-expiratory pressure; Pmax, maximal airway pressure; RASS, Richmond Agitation–Sedation Scale; RR, respiratory rate; SBP, systolic blood pressure; SD, standard deviation; SOFA, Sequential Organ Failure Assessment; SpO_2_, oxygen saturation as measured by pulse oximetry; V_T_, tidal volume.

**Table 3:** Right and left ventricular systolic and diastolic function.

Right Ventricular Variables	Lower PEEP (*n* = 20)	Higher PEEP (*n* = 24)	Point Estimate of the Difference (95% CI)	* p * Value	Left Ventricular Variables	Lower PEEP (*n* = 20)	Higher PEEP (*n* = 24)	Point Estimate of the Difference (95% CI)	* p * Value
Primary parameter									
Myocardial performance index	0.32 (0.26 to 0.39)	0.38 (0.32 to 0.41)	−0.03 (−0.11 to 0.03)	0.33	Myocardial performance index	0.41 (0.37 to 0.49)	0.45 (0.39 to 0.54)	−0.02 (−0.09 to 0.04)	0.35
Systolic parameters					Systolic parameters				
Tricuspid annular plane systolic excursion (mm)	22 (17 to 25)	20 (18 to 22)	0.0 (−3 to 4)	0.75	Ejection fraction, %,	55 (49 to 58)	59 (51 to 65)	−5.1 (−10.9 to 2.9)	0.23
Global longitudinal strain, %, median (IQR)	−18 (−20 to −11)	−22 (−24 to −16)	4 (−2.1 to 9.9)	0.17	Global longitudinal strain, %	−12 (−19 to −8)	−12 (−15 to 10)	0.2 (−2.7 to 3.2)	0.92
Isovolumetric acceleration, m/s	3.3 (3.1 to 4.1)	3.1 (2.5 to 4.9)	0.0 (−1.4 to 0.7)	0.41	Isovolumetric acceleration, m/s	3.2 (2.1 to 4.7)	3.4 (2.1 to 5.9)	−0.3 (−1.5 to 0.6)	0.49
Systolic maximal velocity, cm/s	12.4 ± 3	12.5 ± 4	−0.1 (−2.4 to 2.2)	0.91	Systolic maximal velocity, cm/s	8.3 ± 2.2	8.3 ± 2.3	0 (−1.4 to 1.4)	0.92
Diastolic parameters					Diastolic parameters				
Early/atrial velocity ratio	1.1 (0.8 to 1.2)	0.9 (0.8 to 1.1)	0.1 (−0.1 to 0.3)	0.53	Early/atrial velocity ratio	0.9 (0.72 to 1.1)	1.0 (0.66 to 1.3)	−0.0 (−0.3 to 0.2)	0.81
Early maximal diastolic velocity, cm/s	11.1 ± 3	12.7 ± 4	−1.6 (−4.1 to 0.8)	0.21	Early maximal diastolic velocity, cm/s	8.7 ± 3.4	8.7 ± 3.1	0 (−1.9 to 2.1)	0.95
E/E′	4.1 (3.3 to 5.6)	4.1 (3.3 to 5.6)	0.6 (−0.5 to 1.6)	0.28	E/E′	8.3 (6.1 to 11.6)	8.6 (6.4 to 10.8)	0.2 (−2.4 to 2.7)	0.82
General parameters					General parameters				
Pulmonary acceleration time, m/s^2^	9.1 ± 4	8.9 ± 3	0.2 (−3.1 to 3.2)	0.93	Cardiac index, l min^−1^ m^2 −1^	2.8 ± 0.9	2.5 ± 0.7	0.3 −0.2 to 0.9)	0.24
Right ventricle/left ventricle diameter ^a^	0.72 (0.61 to 0.81)	0.74 (0.61 to 0.93)	−0.02 (−0.2 to 0.1)	0.75	Eccentricity index	1 (0.8 to 1.1)	0.9 (0.8 to 1.0)	0.05 (−0.07 to 0.21)	0.39

Data are provided as mean ± SD when normally distributed; otherwise, median (IQR) is used. Comparisons are shown with the point estimate of the mean or median difference, 95% CI and two-sided *p* value. ^a^ measured at end-diastole. Abbreviations: CI, confidence interval; SD, standard deviation; IQR, interquartile range; PEEP, positive end-expiratory pressure.

## Data Availability

Requests for the data should be sent to Anna Geke Algera; email address: a.g.algera@amsterdamumc.nl.

## Supplementary Materials

The following information can be downloaded at: https://www.mdpi.com/article/10.3390/jcm11092309/s1: Supplement S1. Echocardiographic parameters.
