# Supplementary material for: Myocardial Function during Ventilation with Lower versus Higher Positive End-Expiratory Pressure in Patients without ARDS
Source: J Clin Med. 2022 Apr 21;11(9):2309. doi: 10.3390/jcm11092309 (PMC9104897; doi:10.3390/jcm11092309)
Supplement: Supplementary file 1 [file jcm-11-02309-s001.zip › jcm-1602882-supplementary.pdf]

Supplement S1. Echocardiographic parameters

The echocardiographic exam started with the parasternal long axis view to collect the left ventricle diameters and left ventricular outflow tract. With the addition of the color Doppler the aortic and mitral valves were assessed and graded for the presence of stenosis/regurgitation (absent, mild, moderate or severe). A valvulopathy was considered as significant if graded as moderate or severe.

Thereafter, with the parasternal short-axis view the left ventricular eccentricity index and the pulmonary acceleration time were collected. The left ventricular eccentricity (ratio of left ventricular antero-posterior diameter to the septo-lateral diameter) was used as an measure of right ventricular pressure overload if the value was greater than 1.0. The pulsed-waved sample Doppler was placed within the pulmonary artery to obtain the pulmonary acceleration time (peak velocity divided by time to reach this peak) as a measure of right ventricular afterload. A lower pulmonary acceleration time indicated a higher pulmonary vascular resistance.

Next, the apical four-chamber view was used to collect both left- and right ventricular dimensions. Acute cor pulmonale (ratio of basal right ventricular end-diastolic diameter to basal left ventricular end-diastolic diameter) was used as a measure of right ventricular volume overload when the value was greater than 0.6. With the speckle tracking technique the right ventricular free wall and left ventricular global longitudinal strain were both calculated, with a more negative value indicating a better systolic function. Global longitudinal strain is relatively load independent measure. Thereafter, with the use of color Doppler the mitral and tricuspid valves were assessed for the presence of stenosis/regurgitation. The pulsed-wave Doppler sample volume was placed at the tips of the mitral valve and tricuspid valve, to obtain the left- and right ventricular early/atrial velocity ratio as a measure of left- and right ventricular diastolic function. Next, with the use of tissue Doppler and the pulsed-waved sample volume placed at the septal annulus (only in cases of insufficient images the sample volume was placed at the left ventricular lateral mitral annulus), the systolic maximal velocity and the early diastolic maximal velocity were collected, as measures of left ventricular systolic and diastolic function, respectively. With tissue Doppler and the pulsed-wave sample volume placed in the lateral tricuspid annulus, the systolic maximal velocity and early diastolic maximal velocity were collected as measures of right ventricular systolic and diastolic function. The myocardial performance index (sum of isovolumetric contraction time to the isovolumetric relaxation time divided by the ejection time) is a measure of both systolic and diastolic function and relatively indepedent of loading conditions. A lower MPI indicates a better myocardial function. The isovolumetric acceleration (maximal velocity of the isovolumetric contraction divided by the time to reach this maximum) is a measure of left- and right ventricular systolic function, and is

less load dependent than traditional measurements of systolic function. The tricuspid annular plane systolic excursion (TAPSE) was obtained with the M-mode in the apical four chamber view with the cursor placed through the right ventricular lateral tricuspid annulus as a measure of the right ventricular systolic function.

Both the apical two-chamber view and four-chamber view were used to calculate the left ventricular ejection fraction with the modified Simpson rule.

Thereafter, the apical five-chamber was obtained with the pulsed-wave Doppler sample volume was placed in the left ventricular outflow tract just under the aortic calve to measure the velocity time integral. Stroke volume (velocity time integral obtained from the apical five-chambers view multiplied with the left ventricular outflow tract radius from the parasternal short axis view) was obtained. Cardiac output (stroke volume multiplied with the heart rate) was also obtained.

Finally, with the patient in supine position and the subcostal view was obtained to collect the diameter and distensibility index of vena cava inferior.
